# Decreasing Bone Resorption by Inducing Anti-Osteoclastogenic IFN-γ and IL-10 Expression in the Spleen Through an Electromagnetic Field on LPS-Induced Osteoporosis Mice

**DOI:** 10.3390/bioengineering12090923

**Published:** 2025-08-27

**Authors:** Myeong-Hyun Nam, Hee-Jung Park, Tae-Woo Kim, In-Ho Lee, Hee-Deok Yun, Zuyu Chen, Young-Kwon Seo

**Affiliations:** Department of Biomedical Engineering, Dongguk University, Goyang-si 10326, Republic of Korea; iis05047@naver.com (M.-H.N.); gnflwldk98@naver.com (H.-J.P.); xodn8876@naver.com (T.-W.K.); lih5537@naver.com (I.-H.L.); drengon1538@gmail.com (H.-D.Y.); zuyuchen25@gmail.com (Z.C.)

**Keywords:** anti-osteoclastogenic, IFN-γ, IL-10, spleen, pulsed electromagnetic field

## Abstract

This study sought to evaluate the inhibitory effect of pulsed electromagnetic field (PEMF) therapy on bone resorption in a mouse model of lipopolysaccharide (LPS)-induced osteoporosis. A total of 40 mice were divided into four groups: control, LPS, LPS + alendronate, and LPS + PEMF. Blood and spleen samples were analyzed using RT-PCR and ELISA, while calvaria and femurs were assessed by micro-computed tomography (CT) and histological analysis. Serum analysis revealed that, compared with the control group, calcium levels in the PEMF group showed no significant difference, but alkaline phosphatase (ALP) levels were significantly increased, whereas tartrate-resistant acid phosphatase (TRAP) levels were significantly decreased. Moreover, blood cytokine analysis showed reduced expression of TNF-α and IL-1β and increased expression of BMP-2 in the PEMF group. Spleen tissue analysis further demonstrated significant upregulation of IFN-γ and IL-10 expression in the PEMF group. Micro-CT confirmed that PEMF inhibited femoral bone loss and promoted bone regeneration in calvarial defects. Histological evaluation with hematoxylin and eosin and Masson–Goldner trichrome staining confirmed enhanced bone formation in both the femur and calvaria. In conclusion, PEMF effectively alleviates bone loss and promotes bone regeneration in LPS-induced osteoporosis. Furthermore, PEMF exhibits anti-osteoclastogenic activity by reducing inflammatory cytokines and enhancing IFN-γ and IL-10 expression in the spleen.

## 1. Introduction

Osteoporosis is a condition characterized by an increased risk of fractures resulting from decreased bone microstructure, reduced bone mass, and increased bone fragility [1]. Severe bone loss results from accelerated bone turnover and reduced bone density, ultimately leading to osteoporosis, which affects approximately 200 million people worldwide [2]. This condition places a significant burden on families and society, as many patients require long-term home care [3]. Given the global aging population, the incidence of fractures in women is projected to increase by 310% by 2050 [4]. Currently, there is no consensus on the most effective drug for osteoporosis treatment. Drugs such as immunosuppressants, corticosteroids, and 5-aminosalicylates have proven valuable in treating various diseases. However, their prolonged use is associated with adverse effects, including fever, hypertension, diarrhea, and kidney dysfunction [5]. Among the currently available therapeutic agents, bisphosphonates are widely used due to their strong inhibitory effect on bone resorption. They have been applied in the treatment or prevention of Paget’s disease, oncogenic osteolysis, oncogenic hypercalcemia, osteoporosis, and rheumatoid arthritis. Additionally, bisphosphonates have been used to manage bone loss in children with cerebral palsy, steroid-induced osteoporosis, Duchenne muscular dystrophy, and idiopathic hypercalcemia [6]. Bisphosphonates are well known to inhibit osteoclast activity and have demonstrated the ability to act on osteoblasts in vitro, suppressing osteoclast recruitment and inducing osteoclast apoptosis in both in vivo and in vitro studies [7]. Despite their efficacy, concerns have been raised regarding the adverse effects of bisphosphonates, such as acute flu-like symptoms (fever, joint pain, and muscle pain), gastrointestinal lesions, and osteonecrosis of the jaw [8]. Therefore, exploring more cost-effective and safer non-pharmaceutical strategies for osteoporosis prevention is essential.

Pulsed electromagnetic field (PEMF) therapy, approved by the U.S. Food and Drug Administration (FDA), is a biophysical treatment that stimulates bone formation. It is widely used as an adjuvant therapy in lumbar and cervical fusion surgeries, particularly in high-risk patients prone to non-union or fracture complications [9]. PEMF therapy is clinically safe, effective, and non-invasive and has garnered increasing attention in recent decades [10]. It is commonly applied in the treatment of bone fractures and musculoskeletal disorders, including osteoarthritis and rheumatoid arthritis [11]. Several studies have reported that PEMF stimulation promotes osteoblast proliferation and mineralization while inhibiting osteoclast maturation and activity [11,12,13]. For example, Jing et al. demonstrated that electromagnetic field (EMF) stimulation prevented bone microenvironment deterioration and correlated with increased bone mineral density (BMD) in animal models of osteoporosis [14]. Similarly, Liu et al. reported that PEMF treatment increased BMD, reduced pain, and improved quality of life without adverse effects [15]. Despite these benefits, the signaling pathways and regulatory mechanisms underlying PEMF therapy for osteoporosis remain unclear, potentially limiting its broader clinical application.

Post-traumatic systemic inflammation has been reported to impair fracture healing in patients with multiple injuries [16,17]. Lipopolysaccharide (LPS), a major component of Gram-negative bacterial cell walls, contributes to bone loss by enhancing bone resorption and inhibiting bone formation, as shown in numerous studies [18]. LPS promotes the production of pro-inflammatory cytokines such as tumor necrosis factor α (TNF-α) and interleukin 6 (IL-6), both of which are key mediators of osteoclast differentiation [19].

Animal models are indispensable for evaluating the efficacy and safety of osteoporosis therapies. Rodent models, in particular, offer unique advantages such as low cost, ease of breeding, and short generation times. Extensive research has documented changes in pro-inflammatory cytokine levels and bone histomorphometry in these models [20], both of which are reliable indicators for developing drugs targeting osteolysis and bone resorption [21]. Furthermore, both animal experiments and clinical studies have demonstrated the therapeutic potential of PEMF in bone-related diseases. However, the mechanisms underlying its effectiveness in osteoporosis driven by systemic inflammation remain poorly understood. Our previous in vitro study revealed that a low-intensity (1 mT) 40 Hz EMF applied for 3 h daily effectively suppressed osteoclast differentiation and activity [22]. Additionally, we confirmed that PEMF at 10 mT and 60 Hz for 30 min promoted neurogenesis in a mouse model of stroke [23]. Based on these findings, we selected a 40 Hz frequency, known to inhibit osteoclast differentiation, and applied an intensity of 10 mT for 30 min in this study.

Therefore, the aim of this study was to investigate the effects of PEMF at 40 Hz and 10 mT on pro-inflammatory cytokines, bone turnover markers, and bone remodeling using an LPS-induced osteoporosis model. Specifically, we evaluated the impact of PEMF on pro-inflammatory cytokine production, tartrate-resistant acid phosphatase (TRAP) activity, spleen expression of anti-osteoclastogenic cytokines, and bone microstructure deterioration assessed by micro-computed tomography (micro-CT) and histological analyses in mice with systemic inflammation.

## 2. Materials and Methods

### 2.1. LPS-Induced Osteoporosis Mouse Model

Experiments were conducted using ICR male mice (6 weeks old, *n* = 40; DBL Co., Ltd., Seoul, Republic of Korea). The experimental protocol was approved by the Institutional Animal Care and Use Committee of Dongguk University (IACUC-2022-078-2). Mice were housed under controlled conditions: temperature (23 ± 1 °C), relative humidity (50–60%), and a 12 h/12 h light–dark cycle, with ad libitum access to standard pellets and clean water. Anesthesia was induced via intraperitoneal injection of tiletamine-zolazepam (Zoletil 50^®^, Virbac, Carros, France) at 50 mg/kg. A linear sagittal incision was made along the midline of the scalp, followed by full-thickness retraction of the skin and periosteum to expose the calvarium. Critical-sized bone defects (diameter = 4 mm) were created in the dorsal calvarial region. The 40 mice (7 weeks old at surgery) were randomly divided into four groups (*n* = 10 per group): (i) sham-operated control group (phosphate-buffered saline (PBS, Takara, Kusatsu, Japan) injection: CTRL), (ii) LPS-induced group (5 mg/kg intraperitoneal LPS (Sigma, St. Louis, MO, USA) injection: LPS), (iii) LPS + alendronate group (5 mg/kg LPS intraperitoneally and 1.15 mg/kg alendronate (Sigma, St. Louis, MO, USA) subcutaneously: ALN), and (iv) LPS + PEMF group (5 mg/kg LPS intraperitoneally with PEMF exposure at 40 Hz, 10 mT, 30 min/day: PEMF). LPS was administered intraperitoneally twice per week (every 3–4 days), while ALN was administered subcutaneously once daily [24,25,26]. The experimental protocol was maintained for 17 days. At the end of the study, all mice were sacrificed, and calvarial bones were excised, fixed in 4% paraformaldehyde (PFA, Biosesang, Seongnam, Republic of Korea) for 2 days, and subsequently processed for micro-CT and histological assessment. Spleen tissues were harvested for quantitative reverse transcription polymerase chain reaction (qRT-PCR) analysis.

For the isolation of peripheral blood mononuclear cells (PBMCs), whole blood was collected into EDTA-treated tubes and gently shaken several times to prevent coagulation. Samples were centrifuged at 1000× *g* for 15 min at 4 °C. The upper plasma layer was aspirated and discarded, and the remaining cell pellet was resuspended in 5 mL of ACK lysis buffer and incubated for 5 min at room temperature to lyse red blood cells. To stop lysis, the suspension was transferred to a 50 mL conical tube and diluted with 45 mL of PBS. The solution was centrifuged again at 1000× *g* for 15 min at 4 °C. The supernatant was discarded, and the resulting PBMC pellet was collected for qRT-PCR analysis.

### 2.2. PEMF Exposure System

**Figure 1:**
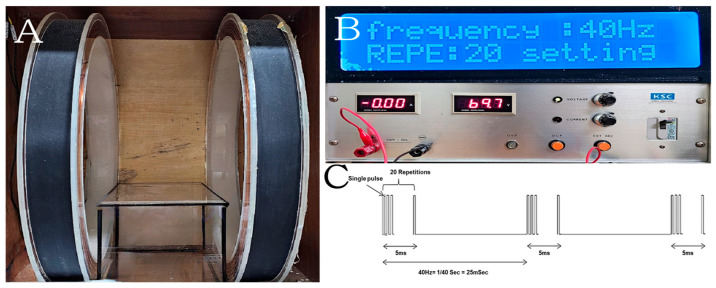
PEMF device used for mouse model treatment, including the generated waveforms and their definitions. The system consists of two main components: solenoid coils (**A**) and a generator for controlling intensity and frequency (**B**). The PEMF signal comprised a 5 ms pulse burst with 20 pulses, repeated at 40 Hz (**C**). The mouse cage was placed between the two solenoid coils and exposed to PEMF stimulation at 10 mT and 40 Hz.

The PEMF exposure system used to stimulate mice consisted of a power amplifier (C130, KOREA SWITCHING, Republic of Korea) and a Helmholtz coil configuration, designed to generate a nearly uniform magnetic field. The system included a pair of identical coils, each 60 cm in diameter, arranged in Helmholtz geometry. The solenoid was configured to deliver PEMFs with a burst frequency of 4 kHz for 5 ms, with a repetition rate of 40 Hz (single burst pulse duration: 0.25 μs, number of repeated single pulses: 20, pulse wait: 20 ms [= 25 − 5 ms]). A continuous PEMF (intensity = 10 mT, frequency = 40 Hz) was applied in this study. The current within the coil was modeled using COMSOL 3. (COMSOL, Inc., Burlington, MA, USA), and the magnetic flux density was measured with a TM-701 Gaussmeter (Tesla meter TM-701, KANETEC, Tokyo, Japan) (Figure 1).

### 2.3. Analysis of Serum Bone Biomarker

After 17 days of PEMF exposure, blood samples were collected from the mice via the basilar artery prior to sacrifice. Whole blood was collected into serum separation tubes (SSTs) (Becton Dickinson [BD], Sunnyvale, CA, USA) and incubated at room temperature for at least 30 min to allow coagulation. Samples were then centrifuged at 1000× *g* for 15 min at 4 °C. The resulting serum supernatant was carefully aspirated, aliquoted, and stored at −80 °C until analysis. Serum markers of bone formation, including calcium (Ca) and alkaline phosphatase (ALP), as well as the bone resorption marker tartrate-resistant acid phosphatase 5b (TRAP), were measured according to the manufacturers’ protocols. Calcium levels were determined using kits from Westang Biological Technology Co., Ltd. (Shanghai, China), while ALP and TRAP were measured using ELISA kits (MyBioSource, San Diego, CA, USA).

### 2.4. Quantitative Analysis of the Gene Expression by qRT-PCR

Total RNA from blood cells and spleen tissue was extracted using TRIzol reagent (Invitrogen, Waltham, MA, USA). To each sample, 200 µL of chloroform (Sigma, St. Louis, MO, USA) was added, thoroughly mixed, and incubated for 3 min. After centrifugation at 12,000× *g* for 15 min at 4 °C, the supernatant was transferred to a new tube and combined with 500 µL of isopropanol. Samples were incubated for 10 min and centrifuged at 12,000× *g* for 10 min, after which the supernatant was discarded. The resulting pellet was washed with 1 mL of 75% ethanol and centrifuged at 7500× *g* for 5 min at 4 °C. The RNA pellet was air-dried at room temperature and dissolved in 20 µL of RNase-free water on ice. RNA concentration was determined using a NanoDrop spectrophotometer (Thermo Fisher Scientific, Waltham, MA, USA).

Complementary DNA (cDNA) was synthesized using a reverse transcription master mix (Dynebio, Seongnam-si, Republic of Korea). qRT-PCR was performed on a StepOnePlus™ real-time PCR system (Applied Biosystems, Waltham, MA, USA) using TB Green^®^ Premix Ex Taq™ (Takara Bio, Kusatsu, Japan). The amplification protocol consisted of an initial enzyme activation step at 95 °C for 20 s, followed by 45 cycles of denaturation at 95 °C for 3 s, annealing at 60 °C for 30 s, and extension at 72 °C for 30 s. After amplification, the melting curve was determined by performing denaturation at 95 °C for 15 s and annealing at 60 °C for 60 s, followed by slow heating in 0.3 °C increments to measure fluorescence and analyze temperature-dependent fluorescence signals. qRT-PCR was used to detect the expression of the osteoblast marker BMP-2 and inflammation-related genes TNF-α, IL-1β, IL-10, and IFN-γ at 17 days after LPS stimulation. All primer sequences are listed in Table 1. Gene expression levels were calculated using the comparative CT (∆∆Ct) method.

### 2.5. Micro-CT

The femur and calvarial bones of each mouse were fixed by immersion in 4% PFA. Both the right and left distal femurs, as well as the calvarial defect sites, were imaged using micro-CT (Quantum FX micro-CT, PerkinElmer, Hopkinton, MA, USA). Scans were performed on the distal femur region and calvarial bone (*n* = 3) under the following parameters: voltage 90 kVp, current 180 μA, scan time 3 min, field of view (FOV) 5 mm, and voxel size 10 µm. Bone parameters were analyzed using Analysis 12.0, including bone mineral density (BMD, mg/cc), bone volume (BV, mm^3^), bone volume fraction (BV/TV, %), trabecular number (Tb.N, 1/mm), and trabecular thickness (Tb.Th, mm).

### 2.6. Hematoxylin and Eosin (H&E) and Masson-Goldner’s Trichrome Staining

The femurs were prefixed in 10% formaldehyde, rinsed in running tap water, and incubated with decalcifying solution (Caci-Clear Rapid™, National Diagnostics, Order No. HS-105). Decalcification was performed at room temperature with continuous shaking, and the solution was replaced every 3 days. The decalcification period was 2 weeks for femurs and 7 days for calvaria. Samples were then washed in distilled water and embedded in paraffin. For histological analysis, two sections (4 µm thick) were cut from each paraffin block using a rotary microtome (Leica RM2255, Wetzlar, Germany). The tissue slices were heated in an oven at 65 °C for 20 min, deparaffinized in xylene for 20 min, and rehydrated through a graded ethanol series (100%, 95%, 85%, 75%, and 50%; 2 min each) before being rinsed in distilled water for 1 min. For H&E staining, longitudinal sections (4 µm) were stained with hematoxylin for 5 min, dipped five times in 1% acid ethanol, rinsed in distilled water, and counterstained with eosin for 3 min. Sections were then dehydrated through graded ethanol and cleared in xylene. For Masson–Goldner’s trichrome staining, slides were treated with Goldner I and II solutions (Carl Roth, Cat. Art.-Nr. 3469.1, 3470.1, Karlsruhe, Germany) for 5 min at room temperature, followed by a 10 min treatment with Goldner III solution (Carl Roth, Cat. Art.-Nr. 3473.1). Stained samples were mounted with Histomount (Agar Scientific, Essex, UK) and examined using an Olympus BX53 fluorescence microscope (Tokyo, Japan).

### 2.7. Statistical Analysis

Data were analyzed using one-way analysis of variance (ANOVA) followed by Dunnett’s post hoc test. Results are presented as mean ± standard error (SE). Differences were considered statistically significant at *p* < 0.05. Significance levels are indicated in the graphs as follows: * *p* < 0.05, ** *p* < 0.01, *** *p* < 0.001, and **** *p* < 0.0001.

## 3. Results

### 3.1. Influence of PEMF on Mouse Body Weight

Table 2 shows the average body weights of mice in each group during the experimental period. Mice in the LPS injection group exhibited a significant decrease in body weight compared with those in the CTRL group at all five time points. In contrast, both the ALN treatment and PEMF exposure groups showed higher body weights than the LPS group. However, the differences in body weight between the ALN or PEMF groups and the LPS group were not statistically significant.

### 3.2. Serum Biochemical Analysis

**Figure 2:**
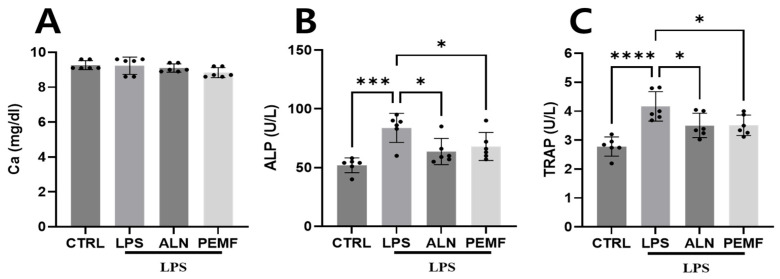
Effects of PEMF exposure on serum biomarkers, including calcium (Ca) (**A**), ALP (**B**), and TRAP (**C**), in an LPS-induced mouse osteoporosis model. Significant differences between groups are indicated as * *p* < 0.05, *** *p* < 0.001, and **** *p* < 0.0001.

The effects of PEMF on bone metabolic biomarkers were evaluated using serum samples collected from mice. As shown in Figure 2, no significant differences in calcium levels were observed among the groups (Figure 2A). ALP levels were significantly higher in the LPS group than in the control group but were significantly reduced in both the ALN and PEMF groups compared with the LPS group (Figure 2B). Similarly, TRAP levels significantly increased in the LPS group compared with the control group and significantly decreased in both the ALN and PEMF groups compared with the LPS group (Figure 2C).

### 3.3. Effect of PEMF on Osteogenic and Inflammation-Related Gene Expression in Blood Cells

**Figure 3:**
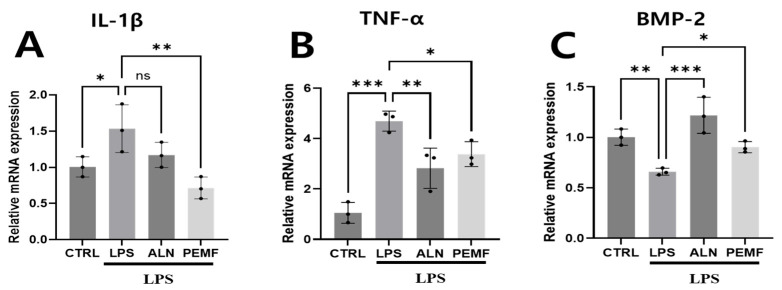
Evaluation of mRNA expression levels of (**A**) IL-1β, (**B**) TNF-α, and (**C**) BMP-2 in blood cells from an LPS-induced osteoporosis mouse model, measured using real-time qRT-PCR. mRNA levels were normalized to GAPDH. Significant differences between groups are indicated as * *p* < 0.05, ** *p* < 0.01, *** *p* < 0.001, and ns indicates not significant.

To evaluate the effect of PEMF exposure on gene expression in blood cells, total mRNA was isolated from all groups and analyzed using qRT-PCR (Figure 3). Gene expression levels were normalized to GAPDH and presented as fold changes. In the LPS group, IL-1β, a marker of early inflammation, and TNF-α, a cytokine involved in bone resorption, were significantly upregulated compared with the control group. In contrast, both the ALN and PEMF groups exhibited significantly lower expression of these inflammatory genes than the LPS group (Figure 3A,B). Expression of the bone formation-related gene BMP-2 was significantly reduced in the LPS group compared with the control group but significantly increased in both the ALN and PEMF groups relative to the LPS group (Figure 3C). In summary, PEMF exposure stimulated the expression of the osteogenic gene BMP-2 while suppressing the expression of the pro-inflammatory genes TNF-α and IL-1β.

### 3.4. Effect of PEMF on Anti-Osteoclastogenesis-Related Gene Expression in Blood Cells and Spleen Tissue

**Figure 4:**
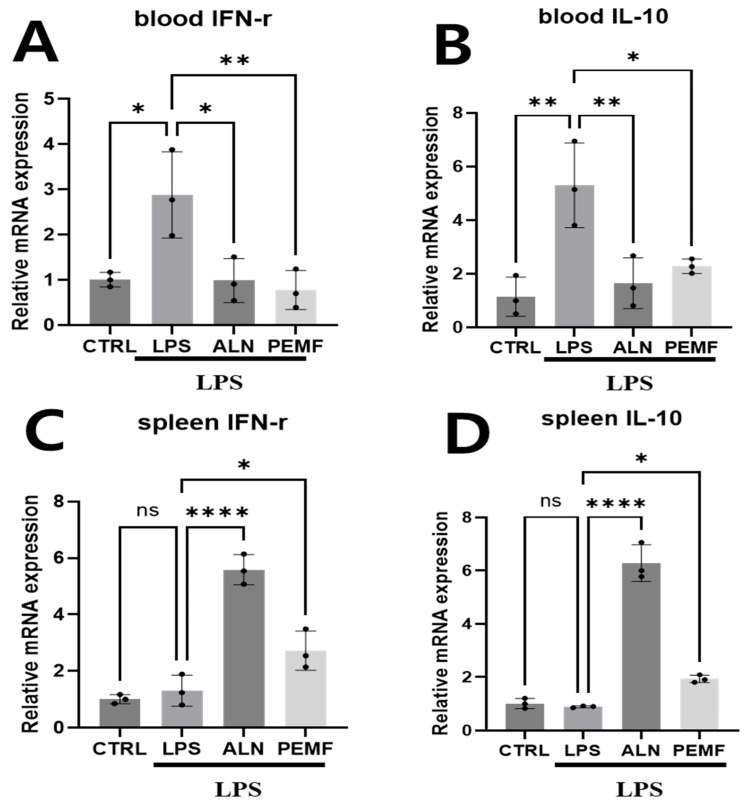
mRNA expression levels of (**A**,**C**) IFN-γ and (**B**,**D**) IL-10 in blood and spleen tissues from an LPS-induced osteoporosis mouse model, analyzed by real-time qRT-PCR. Expression levels were normalized to GAPDH. Significant differences between groups are indicated as * *p* < 0.05, ** *p* < 0.01, **** *p* < 0.0001, and ns indicates not significant.

Figure 4 shows the expression of anti-osteoclastogenesis-related genes in blood cells and spleen tissue. In blood cells, the LPS group exhibited a significant increase in IFN-γ and IL-10 levels compared with the control group, whereas both the ALN and PEMF groups showed significant decreases compared with the LPS group. Although IL-10 expression was higher in the ALN and PEMF groups than in the control group, the differences were not statistically significant (Figure 4A,B). In spleen tissue, IFN-γ and IL-10 expression was markedly elevated in the ALN group compared with the LPS group, with a significant increase also observed in the PEMF group (Figure 4C,D).

### 3.5. Effect of PEMF on Micro-CT and Histological Evaluation of Mice Femur

**Figure 5:**
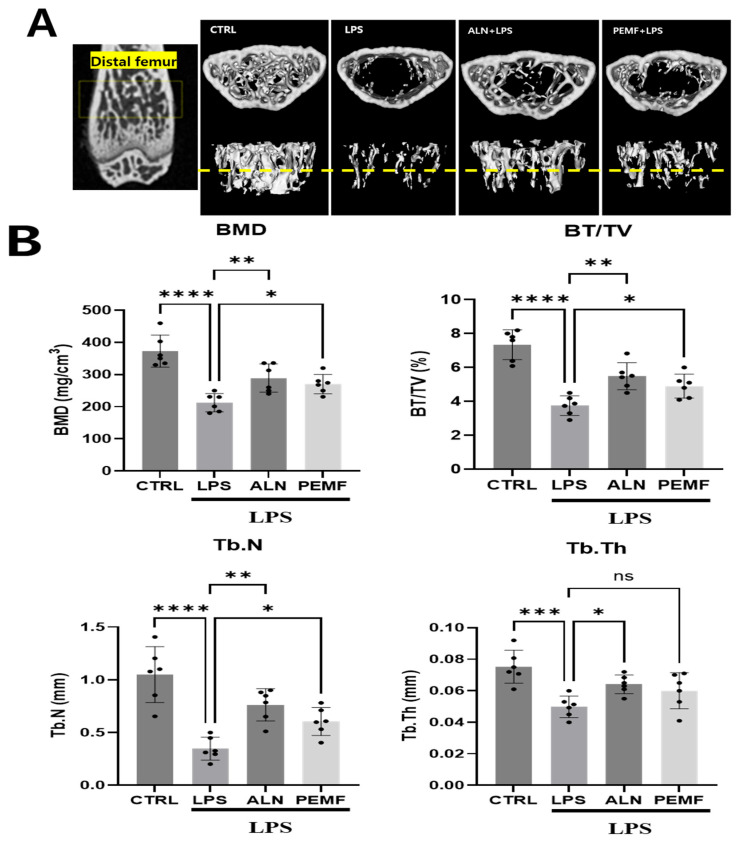
Micro-CT analysis of femurs from an LPS-induced osteoporosis mouse model following PEMF exposure. (**A**) Representative images and (**B**) quantitative analysis of bone mineral density (BMD), bone volume/tissue volume (BV/TV), trabecular number (Tb.N), and trabecular thickness (Tb.Th) are shown. The yellow dotted line indicates the location of the cross-section. Each dot on the graph represents a single sample. Significant differences between groups are indicated as * *p* < 0.05, ** *p* < 0.01, *** *p* < 0.001, **** *p* < 0.0001, and ns indicates not significant.

**Figure 6:**
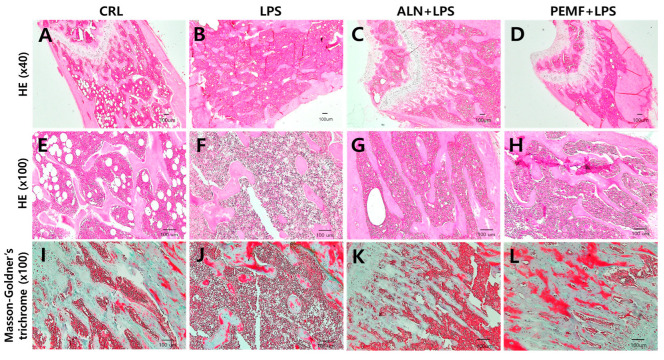
Histological analysis of femurs in an LPS-induced osteoporosis mouse model. (**A**–**D**) H&E staining at ×40 magnification, (**E**–**H**) H&E staining at ×100 magnification, and (**I**–**L**) Masson–Goldner’s trichrome staining at ×100 magnification.

Figure 5A,B show representative micro-CT images and morphometric parameters illustrating the trabecular microarchitecture of the left distal femur across the four groups. Micro-CT analysis revealed significant bone loss in the LPS group compared with the control group, including reductions in trabecular BMD, BV/TV, Tb.N, and Tb.Th. The ALN group prevented trabecular bone loss, showing increases in BMD, BV/TV, Tb.Th, and Tb.N relative to the LPS group. Similarly, the PEMF group exhibited higher BMD, BV/TV, and Tb.N compared with the LPS group. To assess the effect of PEMF on trabecular bone structure, femurs were stained with H&E and Masson–Goldner’s trichrome. As shown in Figure 6, the control group exhibited a well-organized trabecular network with a small bone marrow cavity, whereas the LPS group displayed a disorganized trabecular structure and enlarged marrow cavity. In contrast, the ALN and PEMF groups showed improvements in LPS-induced bone pathology. Masson–Goldner’s trichrome staining, which differentiates tissue by color and morphology, confirmed these observations, with trabeculae identified by green coloration. LPS injection reduced trabecular volume, while ALN treatment and PEMF exposure increased it.

### 3.6. Effect of PEMF on Micro-CT Bone Evaluation of Mice Calvarial Defects

**Figure 7:**
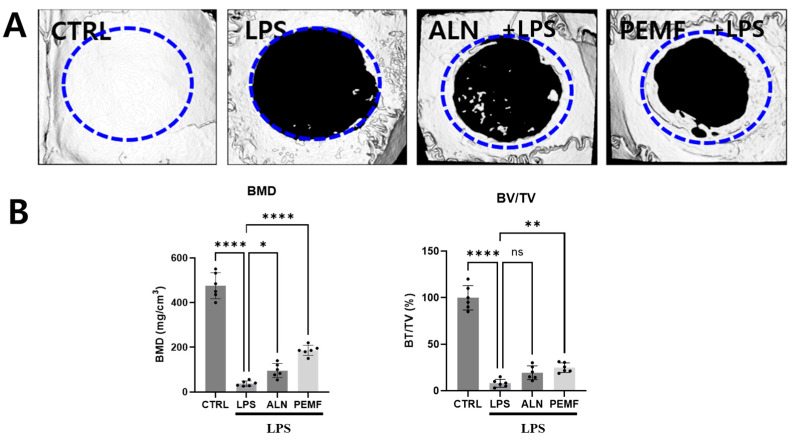
Evaluation of bone regeneration in mouse calvarial defects using micro-CT. (**A**) Top views of reconstructed images; (**B**) quantitative analysis of bone mineral density (BMD) and bone volume/tissue volume (BV/TV) in the defect areas. Significant differences between groups are indicated as * *p* < 0.05, ** *p* < 0.01, **** *p* < 0.0001, and ns indicates not significant.

**Figure 8:**
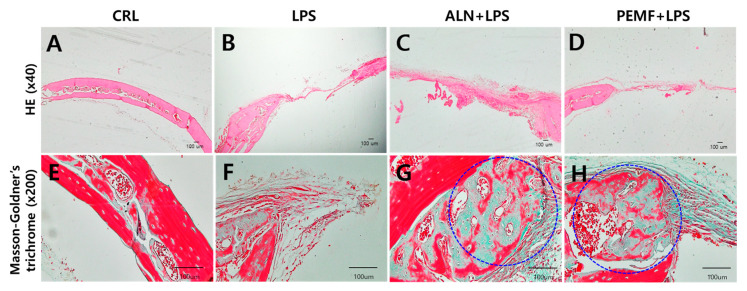
Representative images of calvarial defects in an LPS-induced osteoporosis mouse model. (**A**–**D**) H&E staining at ×40 magnification; (**E**–**H**) Masson–Goldner’s trichrome staining at ×200 magnification.

Figure 7 shows the results of micro-CT analysis used to evaluate the bone regeneration effects of PEMF in three groups, excluding the control group, after calvarial defect creation. In the PEMF group, BMD and BV/TV in the defect area were significantly increased compared with the LPS group. In the ALN group, BMD was significantly higher than in the LPS group, while BV/TV showed a slight, non-significant increase. Histological analysis (Figure 8) revealed no new bone formation in the LPS group. In contrast, newly formed collagen (green, blue circle) was observed in the ALN and PEMF groups using Masson–Goldner’s trichrome staining.

## 4. Discussion

Osteoporosis, characterized by progressive bone loss, represents a major public health concern due to its association with increased fracture risk. Its onset is primarily driven by an imbalance in bone remodeling. Bisphosphonates are potent inhibitors of bone resorption and are widely prescribed for their ability to prevent both vertebral and non-vertebral fractures [27,28]. Despite their effectiveness, prolonged use of these drugs can lead to severe side effects, including esophageal ulcers, muscle and joint pain, headaches, impaired renal function, and osteonecrosis of the jaw [29]. These limitations highlight the need for safer and more effective therapeutic strategies for osteoporosis.

Chronic inflammation, marked by elevated levels of pro-inflammatory cytokines, disrupts skeletal homeostasis by affecting both osteoblast and osteoclast activity, resulting in accelerated bone loss, particularly under conditions of estrogen deficiency [30]. Therefore, modulating pro-inflammatory cytokines is crucial for preventing bone loss and treating osteoporosis.

Serum biochemical markers such as Ca, ALP, and TRAP levels are pivotal serum biochemical markers for bone turnover assessment, indicative of both bone formation and absorption processes [31,32]. Calcium is a key structural component of bone and is commonly used as a marker of bone mineralization [33]. Previous studies in ovariectomized rats have reported either no change or a significant decrease in serum calcium levels [34,35]. In our study, no significant differences in serum calcium were observed among groups, suggesting that PEMF did not alter serum calcium levels. ALP serves as a diagnostic marker for various bone disorders, including rickets, osteomalacia, and Paget’s disease, and its elevated levels are associated with bone and liver pathology [36]. LPS-induced inflammation increases serum ALP as a marker of bone resorption [30]. Previous studies have reported that total ALP levels can serve as an indicator of the efficacy of osteoporosis treatments [37]. Lei et al. reported a significant increase in ALP concentration in an ovariectomy model, which was markedly reduced following PEMF exposure [38]. Similarly, our study demonstrated that PEMF exposure decreased serum ALP levels in an LPS-induced inflammatory osteoporosis model, consistent with findings in the OVX model.

TRAP, secreted by osteoclasts during bone resorption, plays a critical role in bone turnover, and its overexpression can lead to a mild osteoporosis phenotype [39]. Previous studies have shown that TRAP levels sharply increase under conditions simulating osteoporosis in animal models [40,41]. In our study, the LPS-induced elevation of TRAP was attenuated by both ALN treatment and PEMF exposure, suggesting that PEMF can reduce osteoclastogenesis. Nam et al. reported that EMF irradiation inhibited osteoclastogenesis in Raw 264.7 cells via the TRPV4 and p-CREB pathways in vitro [22]. Other studies have shown that EMF reduces reactive oxygen species, thereby suppressing osteoclast differentiation in vitro [42]. Although in vitro studies on the inhibition of osteoclast formation in these electromagnetic fields have been reported [22,42], in vivo evidence for TRAP reduction by EMF has been lacking. In this study, PEMF exposure effectively decreased serum TRAP levels in vivo, providing strong evidence that PEMF can mitigate bone loss by inhibiting osteoclast differentiation.

We further assessed the effects of PEMF on inflammatory cytokine and bone formation-related gene expression in blood cells using an LPS-induced mouse model. LPS is known to induce bone resorption by enhancing inflammatory cytokine production, promoting osteoclast differentiation and activity, and inhibiting osteoblast function [43,44,45,46]. Our results revealed that PEMF exposure mitigated the LPS-induced inflammatory response, as evidenced by a significant reduction in pro-inflammatory cytokines such as IL-1β and TNF-α. BMP-2, a member of the transforming growth factor β (TGF-β) family, promotes osteoblast differentiation by inducing mesenchymal cells to become chondrocytes and accelerating bone repair [47]. LPS administration markedly suppressed BMP-2 expression, whereas PEMF exposure maintained BMP-2 levels comparable to healthy controls. In line with our observations, other studies have reported that PEMF increases BMP-2 expression in disuse osteoporosis models [48]. These findings suggest that PEMF can enhance BMP-2 expression across different osteoporosis conditions. Overall, PEMF suppresses pro-inflammatory cytokines such as TNF-α and IL-1β, reducing bone resorption, while promoting BMP-2 expression to stimulate osteoblast differentiation.

The effect of PEMF on the expression of anti-osteoclastogenic genes in blood cells and spleen tissue was also evaluated. Although osteoclast-mediated bone resorption is essential for normal skeletal development and remodeling, excessive osteoclast formation and activation under pathological conditions can contribute to significant bone loss [49,50,51]. Mechanistically, IFN-γ directly inhibits TNF-α-induced osteoclast formation by inducing apoptosis in bone marrow-derived osteoclasts while also promoting osteoblast activity and suppressing osteoclast differentiation [52,53]. Similarly, IL-10 is a potent inhibitor of osteoclastogenesis, acting by downregulating the production of osteoclastogenic cytokines such as TNF-α and IL-1β, and is therefore considered an anti-osteoclastogenic cytokine [54,55]. In our study, IFN-γ and IL-10 mRNA expression in blood cells was elevated in the LPS group but decreased following ALN treatment.

These results appeared to contradict previous reports, prompting us to further analyze IFN-γ and IL-10 mRNA expression in the spleen. The spleen is a well-known site rich in inflammation- and immune-related cells in mice [56]. Related studies have shown that ovariectomized rats experience bone loss, which can be mitigated by splenectomy [57]. In another study, spleen removal in mice inhibited reductions in bone mineral density following tibial fractures compared to normal mice [58]. Based on these findings, we hypothesized that the spleen plays an important role in osteoporosis and thus analyzed IFN-γ and IL-10 expression.

Analysis of spleen tissue revealed increased expression of IFN-γ and IL-10 in the ALN-administered group, consistent with the known efficacy of osteoporosis treatments. As expected, a similar increase was also observed in the PEMF group. These findings suggest that inflammation-related cells migrate to the spleen, and the spleen effectively reflects the response to osteoporosis-associated cytokines induced by LPS. Our results indicate that, in an inflammatory osteoporosis model, examining anti-osteoclastogenic genes in spleen tissue provides more accurate insights than the analysis of blood cells alone. In summary, PEMF exposure mitigated LPS-induced increases in IL-1β and TNF-α while restoring BMP-2 expression, supporting its potential to counteract inflammation-mediated bone loss.

Furthermore, we confirmed that PEMF exposure also influenced the increased expression of anti-osteoclastogenesis markers, such as IFN-γ and IL-10, in spleen tissues. BMD measurement is widely recognized as a standard tool for diagnosing osteoporosis. Among the measured bone parameters, BMD, BV/TV, Tb.N, and Tb.Th are critical micro-CT indicators for evaluating trabecular bone morphology, particularly in animal models of PEMF-modulated bone loss. In this study, the LPS treatment group exhibited significant reductions in BMD, BV/TV, and Tb.Th compared with the control group, indicating bone destruction and loss under chronic inflammatory conditions induced by LPS [59,60,61]. Conversely, BMD, BV/TV, Tb.N, and Tb.Th were significantly improved in both the ALN and PEMF groups compared with the LPS group. Zhou et al. previously reported that PEMF can increase BMD and enhance balance in rat with osteoporosis [62], supporting the potential applicability of PEMF as a treatment for early-stage osteoporosis caused by chronic inflammation.

Additionally, Huang et al. reported that PEMF exposure mitigates bone microstructure deterioration, as observed through histological analysis [63]. Similarly, our histological examination revealed that the LPS group exhibited bone loss and disorganized trabecular structure, whereas the ALN and PEMF groups displayed a trabecular architecture comparable to that of the control group. These findings suggest that both ALN and PEMF treatments reduce LPS-induced inflammation and osteoclast activation, thereby potentially decreasing bone resorption. To further validate trabecular architecture, Masson–Goldner’s trichrome staining was employed, which enhances the contrast between trabecular bone and bone marrow. This staining method, widely used in bone histology, allows clear tissue identification based on distinct coloration and morphological features [64].

## 5. Conclusions

In this study, we used an LPS-induced inflammatory calvarial defect mouse model to investigate the therapeutic potential of PEMF in mitigating bone loss in vivo. Daily exposure to PEMF at 10 mT and 40 Hz for 30 min effectively reduced femoral bone loss, preserved bone microstructure, and promoted bone formation in calvarial defects. PEMF treatment also suppressed pro-inflammatory cytokine expression and positively modulated bone formation biomarkers in the LPS-induced osteoporosis model. Notably, PEMF exposure resulted in greater bone formation than ALN treatment, highlighting its potential as a standalone therapeutic strategy for osteoporosis.

## Figures and Tables

**Table 1:** qRT-PCR primer sequences.

Genes	(5′-3′)	Sequence
IL-1β	Forward Reverse	GCA ACT GTT CCT GAA CTC AAC T ATC TTT TGG GGT CCG TCA ACT
IL-10	Forward Reverse	GCT CTT ACT GAC TGG CAT GAG CGC AGC TCT AGG AGC ATG TG
TNF-α	Forward Reverse	AGG CGG TGC TTG TTC CTC A AGA CAG AAG AGC GTG GTG GC
IFN-γ	Forward Reverse	GAA CTG GCA GAA GAG GCA CT AGA CAG AAG AGC GTG GTG GC
BMP-2	Forward Reverse	AAG AAG CCA TCG AGG ACC TG CAG TTC CAC ATA CAG CAG GC
GAPDH	Forward Reverse	AGG TCG GTG TGA ACG GAT TTG TGT AGA CCA TGT AGT TGA GGT CA

**Table 2:** Body weights in each group after 17 days of PEMF exposure.

Days	Body Weight (g)											
	CTRL (*n* = 10)			LPS (*n* = 10)			ALN + LPS (*n* = 10)			PEMF + LPS (*n* = 10)		
0	31.5	±	1.6	32.0	±	1.6	32.0	±	1.8	31.7	±	1.6
3	33.0	±	1.7	29.5	±	2 ****	30.5	±	1.8 *	30.7	±	2.0 *
7	33.8	±	1.9	30.1	±	1.4 ****	30.6	±	1.7 ***	30.2	±	1.1 ****
10	33.6	±	2.1	30.4	±	2.1 ***	31.3	±	1.3 *	30.6	±	1.2 **
14	33.7	±	1.8	30.6	±	1.9 ***	31.5	±	1.4 *	31.5	±	1.0 **
17	32.9	±	2.2	30.4	±	1.6 **	31.8	±	1.7	31.3	±	1.3

The experimental results were analyzed using one-way ANOVA, followed by Dunnett’s post hoc test. Significant differences between the control and experimental groups are indicated as follows: * *p* < 0.05, ** *p* < 0.01, *** *p* < 0.001, and **** *p* < 0.0001.

## Data Availability

The original contributions presented in this study are included in the article. Further inquiries can be directed to the corresponding author.
